# Acute effects of air pollutants on adverse birth outcomes in Changsha, China: A population data with time-series analysis from 2015 to 2017

**DOI:** 10.1097/MD.0000000000014127

**Published:** 2019-01-18

**Authors:** Lili Xiong, Zenghui Xu, Jie Tan, Hua Wang, Zhiyu Liu, Aihua Wang, Donghua Xie, Fanjuan Kong

**Affiliations:** a Hunan Province Maternal and Children Care Hospital; b Changsha Environment Protection College; c Hunan Province Environmental Monitoring Centre, Changsha, China.

**Keywords:** air pollutants, low birth weight, macrosomia, preterm birth, time-series

## Abstract

Evidence for the acute effects of air pollutants on adverse birth outcomes is not yet conclusive. Furthermore, there are no investigations relating to the association between air pollutants and macrosomia. The aim of this study was to determine the relationship between air pollutants and low birth weight, preterm birth, and macrosomia in Changsha. Time-series analysis, using a generalized additive model was applied. Data about the adverse birth outcomes was collected from 78 midwifery institutions. Air pollution data including SO_2_, NO_2_, particulate matter <10 μm in diameter (PM_10_), particulate matter <2.5 μm in diameter (PM_2.5_), O_3_, CO, and climate data were respectively collected from the Changsha Environmental Protection Agency and the Changsha Meteorological Bureau from January 2015 to December 2017. During the study period, there were 344,880 live births to be studied. In a single pollutant model, for every increase of 10 μg/m^3^ in PM_10_ and PM_2.5_, low birth weight increased by 0.12% (95% confidence interval [CI]: 0.01–0.23%) at a lag 06 and 0.44% (95% CI: 0.35–0.53%) at a lag 3, respectively. Preterm birth increased most by 1.60% (95% CI: 1.41–1.80%) at a lag 2 for every increase of 10 μg/m^3^ in SO_2_. The highest increases in macrosomia associated with a 10 μg/m^3^ increase in air pollutant were 3.53% (95% CI: 3.41–3.64%) for NO_2_ at lag 0, 3.33% (95% CI: 3.05–3.60%) for SO_2_ at lag03. Multi-pollutant models showed that only PM_10_ increased the low birth weight and preterm birth risk effect by 3.91% (95% CI: 3.67–4.12%) and 0.25% (95% CI: 0.14–0.37%). NO_2_ increased macrosomia risk by 4.14% (95% CI: 3.97–4.31%) with a 10 μg/m^3^ increase. There was no association observed between the air pollutants O_3_ and CO and adverse birth outcomes. Pregnant women should also take steps to limit their exposure to high levels of air pollutants during the final weeks of pregnancy.

## 1 Introduction

Preterm birth, defined as <37 weeks of gestations, is the 2nd largest direct cause of child deaths among children under 5 years.^[1]^ Preterm birth is associated with neonatal mortality and morbidity and can cause long-term adverse health consequences in life.^[2]^ Low birth weight (LBW), defined as a fetal birth weight <2500 g, is a most important predictor of neonatal mortality and is associated with higher risk of infant and childhood mortality and other health problems.^[3]^

Preterm birth and LBW weight have gradually become the focus of environmental epidemiology in recent years. A range of global studies have shown that exposure to air pollutants is linked to preterm birth and LBW.^[4–8]^ In China, there is also increasing evidence that exposure to ambient air pollutants is associated with them.^[9–13]^ However, the findings from western countries may not be adaptive to the county of China in which air pollutants have become an alarming problem coincident with rapid industrialization and urbanization over recent years. In addition, the relevant researches in China showed inconsistent conclusions on the association between air pollutants and preterm birth and LBW.

Macrosomia, defined as a fetal birth weight equal to or greater than 4000 g, irrespective of gestational age, is a serious public health problem worldwide due to its increasing prevalence and adverse influences on maternal and neonatal outcomes.^[14,15]^ The causes of macrosomia are complex, inconclusive, and difficult to interpret.^[14]^ However, no previous study has examined the association between maternal exposure to air pollutants and macrosomia. Studies have proved that air pollutants can change endothelial function, trigger inflammation, and insulin resistance, and are also associated with an elevated risk of hypertension.^[16,17]^ Air pollutants may also adversely affect blood lipid levels, which in turn, may influence blood pressure.^[18,19]^ Maternal serum triglyceride and high-density lipoprotein cholesterol levels at late gestation were related to macrosomia in women without diabetes mellitus.^[20]^ What is more, a recent systematic review of 200 previously published studies showed that air pollution was associated with a greater risk of type 2 diabetes mellitus.^[21]^ Similarly, ongoing research suggests that exposure to air pollution during pregnancy may be related to abnormal glucose regulation and the incidence of gestational diabetes mellitus among pregnant women.^[22,23]^ Maternal diabetes mellitus is one of the risk factors for macrosomia. These results indicated that there is association between air pollutants and macrosomia. To confirm our speculation, our present study demonstrated the association between air pollutants and macrosomia and addressed more than just LBW and preterm birth.

Daily time-series analysis is commonly used to evaluate the short-term effects of air pollutants and adverse birth outcomes.^[9,24,25]^ Time-series studies offer the additional advantage of being able to establish associations in which individual exposure factors that remain unchanged over time pose no bias.^[26]^ Furthermore, no similar studies previously published have used actual population data. Therefore, our study was based on specific population data sourced from 78 midwifery medical facilities in Changsha city in Hunan province.

The characteristics of air pollutants vary in different ways across different regions. Changsha, the capital city of Hunan province, is the economic, financial, cultural, and educational center of Hunan province. Although Changsha has experienced serious air pollution over the past few years as a result of drastic urbanization and industrial expansion, a number of appropriate measures such as low-carbon transportation and a legislation put to ban the use of fireworks for entertainment in rural and urban areas. Most previous studies focused on heavily polluted areas, such as Shanghai and Guangzhou.^[9,11]^ Associations between air pollutants and health topics in the central cities of China have received far less research attention. Therefore, this study aimed to evaluate the risk of adverse birth outcomes and its relationship with air pollutants in Changsha based on population data between 2015 and 2017.

## 2 Materials and methods

### 2.1 Air quality and meteorologic data in Changsha city

Changsha is located in the middle of China (28°12′N, 112°59′E), the capital of Hunan province, with an area of 11,820 km^2^ and a population of 7.31 million. Changsha has a subtropical humid monsoon climate.

The daily concentrations of ambient air pollutants including sulfur dioxide (SO_2_), nitrogen dioxide (NO_2_), particulate matter <10 μm in diameter (PM_10_), particulate matter <2.5 μm in diameter (PM_2.5_), ozone (O_3_), carbon monoxide (CO) from 2015 to 2017 were obtained from Changsha Environment Protection Bureau. There are 12 fixed monitoring stations distributed in 9 administrative areas in Changsha, which collect 24-hour average concentration for NO_2_, SO_2_, PM_10_, PM_2.5_, CO, and 8-hour mean concentrations of O_3_ from 10:00 to 18:00. All of the air pollutants measured by the unit of milligrams per cubic meter (mg/m^3^), except CO, which was measured in the unit of micrograms per cubic meter (μg/m^3^). Daily meteorological data (temperature, atmospheric pressure, wind speed, and relative humidity) during the same period were obtained from the Changsha Meteorological Bureau. Daily values for temperature, atmospheric pressure, wind speed, and relative humidity were calculated by averaging 24 hourly monitoring data.

### 2.2 Data collection for birth outcomes

**Figure 1:**
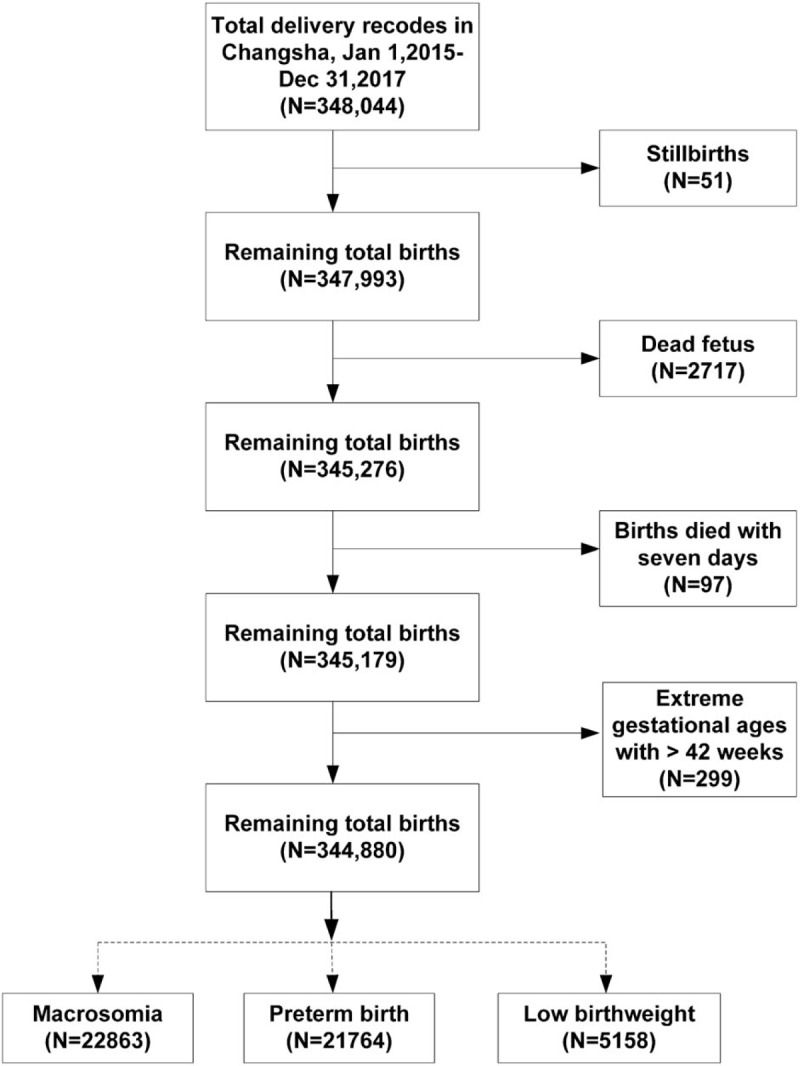
Flow chart showing how the study population was selected.

The delivery information for pregnant women in all midwifery institutions in the Changsha area (N = 78) was recorded in an electronic system developed by Hunan Province Maternal and Children Hospital. Because the medical certificate of birth for every new-born was based upon birth record information in the system, all information was complete and correct. Using these records, we collected a range of data for all new-borns during our study period, including birth weight, the number of gestational weeks at delivery, the birth outcomes (live births, stillbirths, and deaths within 7 days). To facilitate comparisons between the results of our study and those published in literature, we defined a birth weight <2500 g from the live births as LBW, a birth weight more than 4000 g as macrosomia, and a gestational week at delivery of 37 weeks as preterm birth. We had an initial pool of 348,044 birth records in Changsha city between January 1, 2015 and December 31, 2017, which included the permanent delivery pregnant women in Changsha. Further exclusions were made for stillbirths (N = 51), dead fetuses (N = 2717), deaths occurring within 7 days of birth (N = 97), and extreme gestational ages above 42 weeks (N = 299). Ultimately, we had 344,880 births to include in our final analyses (Fig. 1).

The study protocol was reviewed and approved by the Health Department of Hunan Province and the Institutional Review Board at Hunan province maternal and children hospital (2017-S010).

### 2.3 Statistical analysis

We used Microsoft Excel to establish our database. Poission regression, using a generalized additive modeling technique, was used to analyze the associations between daily mean ambient air pollutant concentration and daily birth outcomes. The model was defined by the following equation: 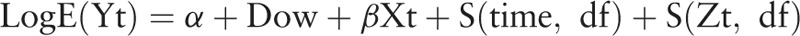


In this equation, *t* refers to the day of the observation; *Y*_*t*_ is the observed daily birth outcome counts on day *t*; *E*(*Y*_*t*_) is the expected daily birth outcomes count on day *t*; α is the intercept; Dow is dummy variable for day of the week; β represents the regression coefficient for each air pollutant; *X*_*t*_ represents air pollutant concentrations at day *t*; *S* is the smoothing spine function for nonlinear variables and *Z*_*t*_ represents meteorologic data at day *t*. The degrees of freedom were then selected according to the minimum value of the Akaike information criterion.^[27]^ In the final model, *S* (time, df) with 5 degrees of freedom for time was used to adjust the time trend,^[28]^*S* (*Z*_*t*_, df) with 3 degrees of freedom were used for all meteorological factors to adjust the effects of whether. The lag effects of air pollutants on birth outcomes were explored from the current day (lag 0) up to 7 days before (lag 7). We also used 2-day to 8-day (from lag 01 to lag 07) moving mean values of air pollutant concentrations to further describe the association. Additionally, to evaluate the stability of pollutant effects, a multi-pollutant model was adopted to assess the confounding effects for all pollutants. All statistical analyses were performed with R V.3.4.3 using the MGCV package (version V.1.8–17, http://www.r-project.org). All results were presented as the percentage change in the relative risk (RR) of birth outcomes along with 95% confidence intervals (CIs) in association with a 10-μg/m^3^ increase in daily air pollutants.

## 3 Results

### 2.4 Description of data

Table 1 shows the summary statistics of daily birth outcomes, air pollutants, and meteorologic data from January 1, 2015 to December 31, 2017 in the Changsha area. During the study period, there were 4 LBW newborns, 21 premature births and 29 live births with macrosomia delivered every day. The daily mean concentrations of NO_2_ ranged from 11 to 109 μg/m^3^, with a mean concentration of 34 μg/m^3^. The daily mean concentrations of SO_2_ ranged from 4 to 71 μg/m^3^, with a mean concentration of 14 μg/m^3^. The daily mean concentrations of PM_10_ ranged from 4 to 338 μg/m^3^, with a mean concentration of 65 μg/m^3^. The daily mean concentrations of PM_2.5_ ranged from 3 to 263 μg/m^3^, with a mean concentration of 46 μg/m^3^. The daily mean concentrations of O_3_ ranged from 6 to 230 μg/m^3^, with a mean concentration of 80 μg/m^3^. The daily mean concentrations of CO ranged from 0.4 to 2.3 mg/m^3^, with a mean concentration of 0.9 mg/m^3^. The mean daily air pressure, wind speed, relative humidity, and temperature were 1001.5 kPa, 2.65 m/s, 80.96% and 17.52°C, respectively.

**Table 1:**
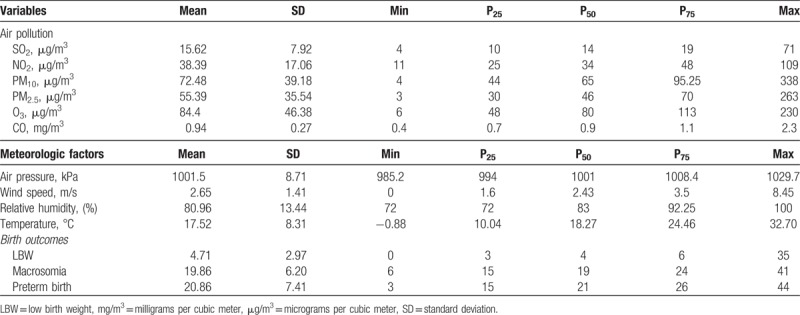
Descriptive summary of daily air pollution, meteorological factors and birth outcomes in Changsha, China, between 2015 and 2017.

### 2.5 Spearman correlation

Table 2 shows the Spearman rank correlation coefficients between air pollutants and meteorologic factors in Changsha during the study period. Wind speed, relative humidity, and temperature were negatively correlated with the 6 air pollutants. Of the 6 air pollutants, NO_2_, SO_2_, PM_10_, and PM_2.5_ were strongly correlated with each other, with PM_10_ and PM_2.5_ showing the highest correlation coefficient of 0.893. All correlations were statistically significant (*P* < .01) except the correlation between PM_2.5_ and O_3_, CO and relative humidity, and SO_2_ and temperature (*P* > .05).

**Table 2:**
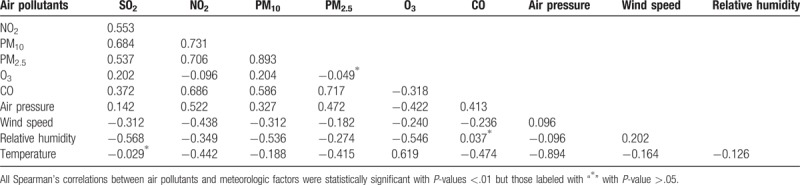
Spearman correlation between air pollutants and meteorologic factors in Changsha, China, between 2015 and 2017.

### 2.6 Time-series analysis

Table 3 shows the increased risk and associated 95% CIs (at different lags) of 3 birth outcomes with every 10 μg/m^3^ increase of each air pollutant; these were evaluated by controlling the influence of meteorologic factors and day of the week. The highest increases in LBW associated with a 10 μg/m^3^ increase in air pollutants were 0.44% (95% CI: 0.35–0.53%) for PM_2.5_ at lag 3. The highest increases in preterm birth associated with a 10 μg/m^3^ increase in air pollutants were 1.60% (95% CI: 1.41–1.80%) for SO_2_ at lag 2. For overall macrosomia, the highest increases in macrosomia associated with a 10 μg/m^3^ increase in each air pollutant were 3.53% (95%CI: 3.41–3.64%) for NO_2_ at lag 0, 3.33% (95% CI: 3.05–3.60%) for SO_2_ at lag 03, 0.37% (95% CI: 0.33–0.41%) for PM_10_ at lag 6, 0.64% (95% CI: 0.60–0.68%) for PM_2.5_ at lag 6.

**Table 3:**
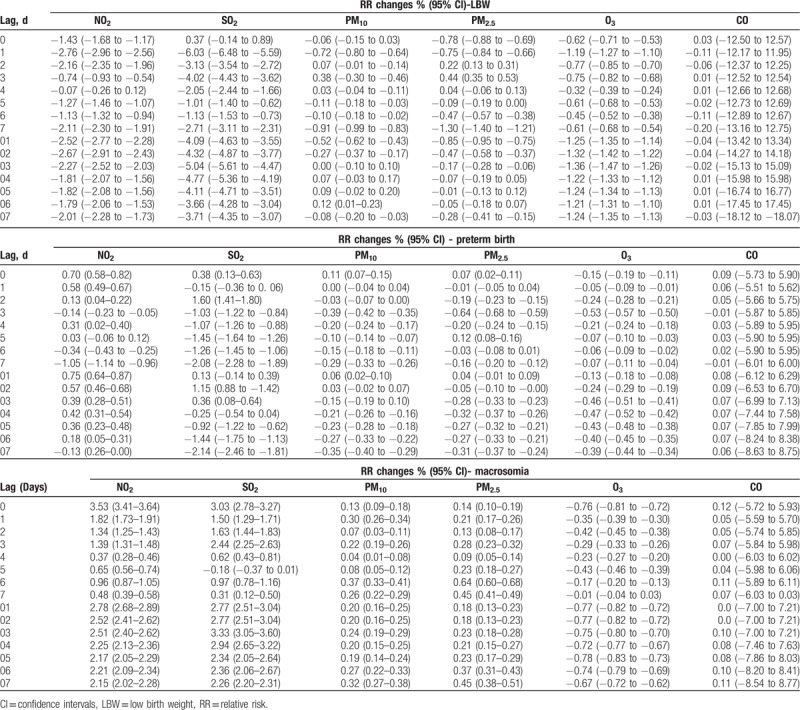
Increased risk change and 95% confidence intervals (CIs) for each 10 μg/m^3^ increase in air pollutants showing associations between air pollutants and birth outcomes.

Table 4 summarizes the increased risk and associated 95% CIs of 3 birth outcomes in multiple pollutant models. In terms of LBW and preterm birth, only PM_10_ increased the risk effect by 3.91% (95% CI: 3.67–4.12%) and 0.25% (95% CI: 0.14–0.37%), respectively, with a 10 μg/m^3^ increase. However, NO_2_ increased the risk of macrosomia by 4.14% (95% CI: 3.97–4.31%) with a 10 μg/m^3^ increase in the multiple pollutant model.

**Table 4:**
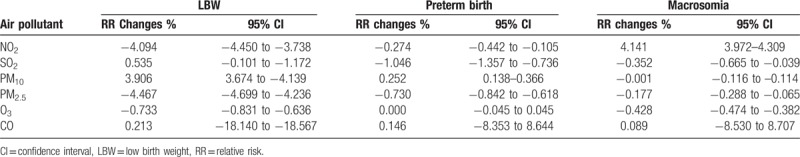
Association between a 10 μg/m^3^ increases in air pollutants with birth outcomes using an all pollutants model for data collected between 2015 and 2017.

## 4 Discussion

This study explored the association between birth outcome data (LBW, preterm birth, and macrosomia) obtained from Hunan Province Maternal and Children Hospital and a range of air pollutants (NO_2_, SO_2_, PM_10_, PM_2.5_, O_3_, CO) in Changsha, China, between 2015 and 2017, using a time-series model. Our findings showed that there was a weak positive association between LBW and short-term exposure to particulate matter. NO_2_, SO_2_, PM_10_, and PM_2.5_ were weakly but positively associated with preterm birth. There was an association between macrosomia and cumulative exposure from 0 to 3 days before birth to NO_2_ and SO_2_ pollution in the ambient air and an association between macrosomia and particulate matter especially at lag 6 to lag 7 days. Furthermore, 2- to 8-day (from lag 01 to lag 07) moving mean values of NO_2_, SO_2_, PM_10_, and PM_2.5_ concentrations were all associated with an increased risk of macrosomia. However, there was no association between O_3_ and CO and adverse birth outcomes. PM_10_ increased the risk of LBW and preterm birth in all pollutant models, while NO_2_ increased the risk of macrosomia in all pollutant models. This represents the 1st study to investigate the association between air pollutants and birth outcomes, including macrosomia, in Changsha.

The daily mean concentration of PM_2.5_ and PM_10_ was higher than the national ambient air quality standard (NAQS) primary standard, but lower than the secondary standard accordingly. The daily mean concentrations of SO_2_, NO_2_, O_3_, and CO were all lower than their respective NAQS primary standard. The daily mean concentrations of PM_10_, SO_2_, and NO_2_ in Changsha area between 2015 and 2017 were lower than the PM_10_ (105.91 μg/m^3^), SO_2_ (20.57 μg/m^3^), NO_2_ (30.93 μg/m^3^) in Hefei city between 2010, and 2015. Hefei city is the capital of Anhui province and is located in the east of China (31°52′N, 117°17′E).^[29]^ Similarly, the daily mean concentrations of PM_10_, SO_2_, and NO_2_ in Changsha were lower than the PM_10_ (115.60 μg/m^3^), SO_2_ (53.21 μg/m^3^), NO_2_ (53.08 μg/m^3^) in Wuhan city between 2006 and 2009. Wuhan city is the capital of Hubei province and located at the center of China (30°33′N, 114°19′E).^[30]^ Although economic development has been achieved at the expense of the environment over the last few years, the government has been working hard to come up with policies and measures to tackle air pollution. Therefore, the concentration of air pollutions is starting to decrease. Reducing the concentration of particulate matters is a specific target for the Changsha government in the future.

In the present study, we found that air pollutants were negatively associated with LBW in single pollutant models except for PM_10_ at lag 06 day and PM_2.5_ at lag 2 and lag 3 days. PM_10_ showed the highest risk of LBW in the all pollutant model. A previous study, involving 22 countries in the World Health Organization Global Survey on Maternal and Perinatal Health from 2004 to 2008, found higher PM_2.5_ levels were associated with a higher risk of LBW; this relationship was identified by using generalized estimation equations.^[31]^ A total of 23 studies published before July 2016 were collected and analyzed; the authors concluded that PM_2.5_ exposure throughout pregnancy may increase the risk of term low birth weight.^[32]^ In this present study, our results showed that exposure to particulate matters increased the risk of LBW. A plausible explanation for this might be that a low placenta weight is related to birth weight during pregnancy; the placenta is a vital organ as it supports the nourishment, growth and development of the embryo.^[33]^ During pregnancy, exposure to maternal particulate matter may represent an important risk factor for intrauterine inflammation which could then affect the growth, development, and function of the placenta.^[34]^ In this present study, a weak positive association between short-term exposure to particulate matters and LBW was observed for the reason of low concentration. Additional research is now needed to gain a better understanding of the impacts of air pollution on LBW, including the identification of susceptible sub-populations, the effects of multiple pollutants, and the effect of different types of weather, time periods, and differential study designs.^[35]^

In the present study, we found that NO_2_ was associated with preterm birth in single pollutant models at lag 0 to lag 2 days, lag 4 and lag 5 days, and lag 01 to lag 06 days. SO_2_ was associated with preterm birth at lag 0, lag 2, and lag 01 to lag 03 days in single pollutant models. PM_10_ was associated with preterm birth at lag 0 and lag 01 days in single pollutant models, while PM_2.5_ was associated with preterm birth at lag 0 and lag 5 days. There was no association between O_3_ and CO and preterm birth in our single pollutant models. Previous studies regarding the specific pollutants linked to preterm birth have been very inconsistent. A study in the Middle East, which took place between 2015 and 2018 showed that significant relationship between each 10-unit increase in NO_2_ and CO, and premature birth in lag 0; this relationship was identified by conducting a time-series study adjusted by trend, seasonality, temperature, relative humidity, weekdays, and holidays.^[36]^ Another study conducted a time-series analysis of metropolitan Atlanta between 1994 and 2004 and showed that PM_2.5_ was associated with preterm birth in the final week of gestation.^[37]^ One large study of London, covering 13 years between 1988 and 2000, and using time-series regression techniques, suggested that there was no association between preterm births and cumulative exposure from 0 to 6 days before birth to ambient air pollution of PM_10_ and O_3_ or recent changes in the weather.^[25]^ In another study, Zhao et al reported a 0.7% increased risk of preterm birth associated with each 10 μg/m^3^ increase in PM_10_ on day 4 of the week before delivery in Cuangzhou, China.^[10]^ Evidence also suggests that inflammatory pathways, as well as implantation errors in early pregnancy, play a role in preterm birth, although the pathophysiology of preterm birth remains poorly understood.^[2]^ Air pollutants could increase the risk of preterm birth by affecting these 2 pathways. The levels of air pollutants in the weeks following conception could disrupt implantation and placentation, and the high levels of air pollution during late pregnancy could activate either an acute or sustained inflammatory response, thus leading to the initiation of early labor.^[37]^ Most of our present results showed a weak relationship between air pollutants and preterm birth. This may have been related to that we investigated ambient concentrations in the preceding week of birth and not the weeks following conception. We found the increased risk of low birth weight and preterm birth associated to cumulative exposure to PM_10_ is not significant in a single pollutant model if compared to the increased risk detected in the multi-pollutant model. A study in Hefei city had also found similar phenomenon, in which one pollutant showed robust effect after other pollutants entered into the model.^[29]^ It suggested the PM_10_ might be more important in air pollution mixture.

In the present study, we also found that the air pollutants NO_2_, SO_2_, PM_10_, and PM_2.5_ were associated with macrosomia in single pollutant models. NO_2_ and SO_2_ were associated with macrosomia at lag 0 to lag 7 days and lag 01 to lag 07 days, except for SO_2_ at lag 5. NO_2_ showed the highest risk of macrosomia in our all pollutants model. However, many studies have reported an association between air pollution and diabetes mellitus. For example, a systematic review in Europe and north America synthesized the results of studies on type 1 and type 2 diabetes mellitus, and gestational diabetes, and showed that PM_2.5_ and NO_2_ increased the risk of diabetes mellitus by 8% to 10% per 10 μg/m^3^ increase in exposure.^[38]^ Available evidence from other systematic reviews and meta-analyses supports a prospective association of NO_2_ and PM_2.5_ with an increased risk for diabetes mellitus.^[39,40]^ This present study found higher levels of NO_2_ to be significantly associated with increasing risk of macrosomia after introducing other air pollutants. Other studies have also observed that the effect of NO_2_ was enhanced when all air pollutants were assessed together.^[41–43]^ The biological mechanisms that link air pollution to the development of macrosomia remain unclear, although one possible explanation for this is that air pollution causes maternal diabetes, which represents a pathological factor which could lead to macrosomia. Another pathway might be the systemic inflammation caused by air pollutants that results in metabolic dysfunction.^[17]^ Furthermore, obesity and over-nutrition, risk factors for the development of diabetes, may render women more susceptible to the effects of air pollution^[44]^ and also promote the development of macrosomia during pregnancy. In future similar studies, it will be necessary to explore the relationship between air pollution and macrosomia after 1st considering maternal diabetes status.

This study had multiple strengths. First, the use of population data allowed for the inclusion of all births in the Changsha area, whereas most previous studies included only components of data from the city being studied. Second, the birth information used in our analyses was all correct and complete because it was linked to the birth certificate, which is a legal document supervised by the public security department. Third, this was the 1st study to examine whether air pollutants are associated with macrosomia.

This study also had some limitations. First, our analyses were not adjusted for infant gender, maternal age, race, maternal smoking status, and maternal health status due to lack of these individual risk factors. Future studies could focus on personal risk factors especially time varying factors such as maternal smoking status and maternal health status to confirm our findings. Second, the potential issue of linearity was not considered in this study; this may have led to some instability in our multiple models. Third, the exposure levels provided by outdoor monitors may not fully represent individual exposure levels. Nonetheless, even a small increase in the risk for advanced birth outcomes could have a major effect on public health following ubiquitous exposure. This concept requires further investigation.

## 5 Conclusion

The results obtained in this study indicated that during the study period, particulate matter was weakly associated with low birth weight and that both SO_2_ and NO_2_ influenced the incidence of preterm birth and macrosomia in Changsha. Despite the low levels of air pollutants in Changsha, pregnant women should make a specific effort to limit their exposure to high levels of air pollutants during the final weeks of pregnancy.

## Acknowledgment

The authors gratefully acknowledge all the members involved in the data collection of delivery information, air quality, and meteorologic data in Changsha city.

## Author contributions

**Conceptualization:** Lili Xiong, Zenghui Xu.

**Data analysis:** Lili Xiong, Zenghui Xu.

**Data collection:** Lili Xiong, Zenghui,Xu, Jie Tan, Zhiyu Liu, Aihua Wang, Donghua Xie, Fanjuan Kong.

**Funding acquisition:** Lili Xiong.

**Methodology:** Lili Xiong, Zenghui Xu.

**Project administration:** Lili Xiong, Hua Wang, Zhiyu Liu.

**Supervision:** Lili Xiong, Zenghui Xu, Zhiyu Liu, Hua Wang.

**Writing – original draft:** Lili Xiong, Zenghui Xu.

**Writing – review & editing:** Lili Xiong, Zenghui Xu, Hua Wang, Zhiyu Liu.
